# Development of Chimeric Ribonuclease A Inhibitor for Molecular Biology Applications: SUMO Fusion as an Engineering Strategy

**DOI:** 10.3390/cimb48060637

**Published:** 2026-06-18

**Authors:** Dmitry Sukhov, Tatyana Petrova, Daria Kruglova, Inna Kholoshenko, German Romanenko, Yuri Utkin, Pavel Pantyushenko, Dmitry Trofimov, Vladimir Korotkiy, Ekaterina Barsova, Yulia Kirillova

**Affiliations:** 1 DNA-Technology LLC, 117587 Moscow, Russia; sukhovdim@yandex.ru (D.S.); t.petrova@dna-technology.ru (T.P.); dasha-sokol-kruglova@yandex.ru (D.K.); innakholos5@gmail.com (I.K.); germanromanenko@rambler.ru (G.R.); d.trofimov@dna-technology.ru (D.T.); 2 M.V. Lomonosov Institute of Fine Chemical Technologies, MIREA–Russian Technological University, 119454 Moscow, Russia; sarov.vova0409@gmail.com (V.K.); dryulets@mail.ru (Y.K.); 3 Shemyakin-Ovchinnikov Institute of Bioorganic Chemistry, Russian Academy of Sciences, 117997 Moscow, Russia; barsova.kat@gmail.com; 4 Zelinsky Institute of Organic Chemistry, Russian Academy of Sciences, 119991 Moscow, Russia; pantyushenko.pavel@gmail.com; 5 Department of Molecular Technologies, Pirogov Russian National Research Medical University, 117997 Moscow, Russia

**Keywords:** RNase inhibitor, SUMO fusion, protein engineering, chimeric protein, RNA stability, protein–protein interaction, RT-qPCR

## Abstract

A chimeric RNase A inhibitor (SUMO-RI) was produced by fusing a SUMO domain to the N-terminus of the murine Rnh1 protein. Functional assays demonstrated that SUMO-RI effectively protects RNA from RNase A-mediated degradation under conditions mimicking real-time RT-PCR, with performance comparable to that of commercial RNase inhibitors. The primary advantage of the chimeric design is its improved technological suitability: SUMO-RI exhibits markedly enhanced storage stability relative to the recombinant Rnh1 inhibitor. However, this benefit comes with a trade-off—SUMO fusion reduces thermostability at temperatures above approximately 47 °C. Together, these findings establish SUMO fusion as a rational engineering strategy for RNase inhibitors, offering improved practical handling at the expense of thermal resilience.

## 1. Introduction

Modern molecular diagnostics relies heavily on the sensitivity and specificity of the systems and materials used to analyze samples, and the careful preservation of target nucleic acids is key to the testing process. Ribonucleic acid (RNA), a key biomarker of gene expression and a direct substrate for the detection of RNA-containing viruses such as SARS-CoV-2 and hepatitis C, is extremely vulnerable to hydrolytic degradation by ubiquitously present ribonucleases (RNases). Even trace amounts of these enzymes can lead to irreversible degradation of the target RNA, which jeopardizes the reliability of a range of methods, including real-time reverse transcription-polymerase chain reaction (RT-PCR), new generation sequencing (NGS) and isothermal amplification. To combat this, the development of highly effective RNase inhibitors (RIs) which are stable over time and at different temperatures is a priority in bioanalytics and biotechnology.

Ribonuclease inhibitor (RI), also known as Rnh1, is a leucine-rich repeat protein that binds to pancreatic-type RNases with femtomolar affinity [1,2]. Structural studies have revealed that RI adopts a horseshoe-shaped architecture, and the complex with RNase A involves an extensive interface dominated by the C-terminal half of the inhibitor [1]. This high-affinity interaction is conserved across species, yet subtle differences in interface geometry and electrostatics determine selectivity toward different RNases [2]. Beyond its canonical role as a cytosolic sentry that neutralizes mislocalized or secretory RNases [3,4], RI has been implicated in regulating angiogenesis, oxidative stress responses, and intracellular redox homeostasis [5,6,7]. Knockout studies have underscored its essential protective function against exogenous RNases [8,9]. The structural and functional diversity of RI–RNase complexes has been characterized for several homologues, including the murine RI–RNase 1 complex (PDB: 3tsr), which serves as a relevant reference for the system studied here [2,10].

The efficient production of recombinant RIs in bacterial expression systems (such as *E. coli*), particularly those based on the murine Rnh1 protein, is associated with a number of fundamental technological problems [2]. These include:

1. The tendency for the inhibitor to form insoluble protein aggregates (inclusion bodies) during production, leading to a significant reduction in the yield of soluble and functionally active Rnh1 protein [11];
2. High redox sensitivity due to the prevalence of cysteine residues, negatively affecting the stability of inhibitors during storage and use [12];
3. The relatively low productivity of the process overall [12].

These limitations significantly complicate the production of reliable and affordable RIs that work effectively under diagnostic protocols [12].

Despite the widespread use of recombinant RIs, innovative engineering approaches aimed at improving the solubility, stability, and functional suitability of these proteins remain limited. One promising production method which aims to address these concerns involves the creation of chimeric constructs, merging the target protein with a helper protein (fusion tag). Tags such as thioredoxin (TRX), maltose binding protein (MBP), glutathione-S-transferase (GST), and, in particular, Small Ubiquitin-like Modifier (SUMO) perform several functions: they increase the solubility and stability of the target protein in vivo, facilitate its purification via metal affinity chromatography, and in the case of SUMO, allow for highly precise specific enzymatic cleavage using SUMO-specific proteases [13].

In recent years, SUMO technology has moved beyond simply increasing solubility. SUMO has been shown to act as a “chaperone mimic”, stabilizing fusion partners through hydrophobic interactions [13,14]. Recent studies have successfully applied SUMO fusions to difficult-to-express proteins, including kinases and membrane proteins [15,16]. However, systematic evaluations of SUMO fusions as standalone functional constructs have not been performed for RIs. Moreover, existing data are contradictory: Guo et al. [17] showed that SUMO was less efficient than MBP, NusA, and IF2 in terms of soluble protein yield, but in that work SUMO-RI was not considered as a final reagent—it was evaluated only as a temporary tag. The present study fills this gap by providing a comprehensive analysis of SUMO-RI—from structural modeling to application in a diagnostic RT-qPCR format.

The data available [17] emphasize the low solubility of RIs when expressed in *E. coli*, and this was one of the key factors affecting the choice of fusion tag for the construction of a chimeric RI. Previous attempts to use SUMO as a fusion partner for murine RI showed that it was less efficient in terms of soluble yield compared to MBP, NusA, or IF2 when the goal was to recover the native inhibitor after tag removal [17]. However, those studies did not evaluate the chimeric SUMO-RI as a standalone functional reagent, nor did they provide a detailed structural rationale for the fusion architecture. Moreover, the technological profile of SUMO-RI—including storage stability, effective temperature range, and performance in diagnostic workflows—remains unexplored. The present work is focused on the SUMO domain, which allowed increased solubility of the resulting chimeric RI despite bacterial expression as well as the simplification of the purification process. Specific proteolytic cleavage of the SUMO domain from the target protein by the protease bdSENP1 also ensured that the N-terminus of the obtained protein Rnh1 would be intact. Unlike most previously published works involving SUMO as a solubilizing tag [13], this study analyzed in depth the effects of the SUMO module on the properties of the resulting RI at the structural, functional and application levels. In this study, Rnh1 was obtained not by independent expression but by enzymatic cleavage of SUMO-RI. We used this approach because attempts to directly express Rnh1 in a soluble form in our system (even with chaperone co-expression) did not yield the protein in the quantity and purity required for comparative analysis.

The present study therefore aims to present not only a method of increasing the technological suitability of RIs, but also presents substantial biochemical evidence to support the continued use of the chosen chimeric architecture for the production of RIs.

To realize these goals, the following steps were achieved:

1. Creation of a genetic construct and production of a recombinant chimeric RNase A inhibitor (SUMO-RI, based on murine Rnh1);
2. Production of the Rnh1 form of the inhibitor via specific proteolytic cleavage of the SUMO domain;
3. Evaluation of the effects of the SUMO module on the technological and functional characteristics of the inhibitor in vitro;
4. Investigation of the structural features of the interaction between the chimeric inhibitor and RNase A;
5. Comparative functional assessment of the inhibitory activity of the chosen RIs: SUMO-RI, the Rnh1 form of the inhibitor and two commercially available RIs;
6. Evaluation of the suitability of the developed inhibitors to conditions simulating in vitro molecular biology and diagnostic protocols.

## 2. Materials and Methods

### 2.1. Materials

Nucleotide sequences of genes were synthesized by GeneQuest (Moscow, Russia). Cultivation of recombinant *E. coli* strains was carried out in a 15 L fermenter (Biotechno, Moscow, Russia), filled with 10 L of nutrient medium. The target product was purified in a Unique GS AutoPure 50 medium-pressure chromatographic system (Good Science Instrument Technology, Shanghai, China), using Cytiva columns (Marlborough, MA, USA) packed with affinity (Bio-Works, Uppsala, Sweden) and ion-exchange (Galak, Beijing, China) resins. All reagents used in the work were of analytical grade or higher. RNase A was from NEB (Monarch^®^ RNase, Ipswich, MA, USA, cat. No. T3018L), protease bdSENP1 and inhibitor of RNase A LoRI were from GeneQuest LLC (Moscow, Russia, cat. # PS-008-001K and cat. # ME-411–001K), and RiboLock RNase Inhibitor (40 U/μL) was from Thermo (Waltham, MA, USA, cat. # EO0381). Synthetic RNA was from SARS-CoV-2/SARS-CoV detection kit (cat. No. R3-P436-S3/9, DNA-Technology LLC, Moscow, Russia) where it served as an internal control.

### 2.2. Structural Modeling

For structural modeling, we took a chimeric SUMO-RI protein in which the sequence of the murine RI (Rnh1; Uniprot accession number Q91VI7) was fused at its N-terminus with a SUMO domain (NCBI Reference Sequence XP_052162678.1). A His-tag was added to the SUMO-RI N-terminus also. Boltz-2, an improved version of the AlphaFold 3 software designed to predict the structures of protein–protein complexes [18], was used to model the complex of chimeric SUMO-RI with RNase A (Uniprot accession number Q9BEC3). This tool was chosen because of its accurate modeling of protein–protein complexes and its ability to assess the quality of its own predictions. The quality of the models was evaluated using the built-in metrics of confidence score, pTM and ipTM. Intermolecular interactions in the predicted complex were analyzed using the PLIP 2025 instrument [19].

### 2.3. Molecular Cloning and Bacterial Expression System

To make the purification of target proteins easier, A His-tag was added to the SUMO-RI N-terminus. The designed SUMO-RI amino acid sequence was converted to DNA sequence and codon-optimized for expression in *E. coli*. SUMO-RI coding DNA was synthesized and cloned into the pET28 plasmid (Novagen, Madison, WI, USA). The correctness of construct sequence (pET28-SUMO-RI) was verified by Sanger sequencing. To enhance the yield of soluble active protein, the expression host *E. coli* BL21(DE3) was first transformed with the chaperone plasmid pGro7 (Takara Bio, Kusatsu, Japan). This plasmid expresses the GroEL/ES chaperone system, which is recommended for folding recombinant proteins rich in cysteine residues [20]. This generated a dedicated producer strain, BL21(DE3)/pGro7. Finally, the BL21(DE3)/pGro7 strain was transformed with the verified pET28-SUMO-RI construct for protein expression.

### 2.4. Bacterial Culture

The producer strain BL21(DE3)-pGro7, transformed by the pET28-SUMO-RI construct, was grown overnight (ON) at 37 °C in 5 mL modified TB medium: 20.0 g/L trypton, 24.0 g/L yeast extract, 12.0 g/L potassium hydrophosphate, 2.0 g/L potassium monophosphate, and 1.0 g/L magnesium chloride at a pH of 7.0 ± 0.2 at 25 °C, with the addition of kanamycin and chloramphenicol to final concentrations of 50 µg/mL and 30 µg/mL, respectively. The culture was then transferred to a fresh portion of 400 mL TB medium with antibiotics and incubated ON in a shaker (225 rpm) at 37 °C. The next day, the culture was diluted with 10.0 L TB medium supplemented with 2.5 mM MgCl_2_ and grown in an F25L bioreactor (BIOTECHNO, Moscow, Russia) at 37 °C, 400 rpm, 0.6 bar and aeration to equal 0.25 m^3^/h to an exponential phase defined as OD_600_ = 0.7. Next, 1.0 g/L arabinose was added to activate the chaperone gene, and bacteria were allowed to grow at 37 °C up to OD_600_ = 1. The temperature was then lowered to 20 °C and the protein production was induced with isopropyl β-d-1-thiogalactopyranoside (IPTG) at a final concentration of 1 mM. Twenty hours after induction, cells were harvested by centrifugation at 4500 rpm in a Beckman Coulter Avanti™ J-15R centrifuge equipped with a Beckman Coulter Avanti™ JS-4.750 rotor (Beckman Coulter; Brea, CA, USA). The Rnh1-only construct was expressed in the same BL21(DE3)/pGro7 host and under identical cultivation and induction conditions as SUMO-RI.

### 2.5. Isolation of SUMO-RI

Isolation and primary purification of the recombinant chimeric inhibitor was carried out using the procedure followed for SUMO-RI [21]. In brief, 60 g of the cellular biomass underwent lysis, after which the target proteins were isolated via ammonium sulfate precipitation and subsequent metal-affinity chromatography on a WorkBeads NiMAC sorbent (BioWorks, Uppsala, Sweden), followed by anion exchange chromatography. The obtained fractions were analyzed in 10% SDS-PAGE and those comprising SUMO-RI were combined and dialyzed against 50 mM Tris-Cl buffer (pH 8.0), containing 150 mM NaCl, 0.1% Tween-20, and 5 mM DTT at a ratio of 1:50 for 16 h.

### 2.6. Isolation of RI

After dialysis, the protease bdSENP1 (GeneQuest LLC, Moscow, Russia, cat. # PS-008) was added to the SUMO-RI solution at a protease:substrate mass ratio of 1:100, and the reaction mixture was incubated at constant stirring (100 rpm) for 16 h at 20 °C. After completion of proteolysis, the mixture was dialyzed against 1 L of 50 mM Tris-Cl buffer (pH 7.0) for 16 h at 4 °C, then underwent metal-affinity chromatography column (Smart-Lifesciences, Changzhou, China) filled with 1 mL WorkBeads NiMAC sorbent, pre-equilibrated with the same buffer. Under these conditions, the cleaved Rnh1 inhibitor, which did not contain a His-tag, eluted unretained from the column, while the SUMO domain with a His-tag was retained on the metal-affinity sorbent. The efficiency of the proteolytic cleavage and separation of components was checked in 10% SDS-PAGE. The fraction containing recombinant RI was dialyzed against a storage buffer (50 mM KCl, 20 mM Hepes-K, 0.1% Tween-20, 8 mM DTT, 50% glycerol; pH 7.2) at a volume ratio of 1:40 for 12 h. The concentration and purity of the resulting preparation were determined spectrophotometrically using the Bradford method with BSA as standard, and with SDS-PAGE, respectively. The resulting stock solution was diluted with a storage buffer (50 mM KCl, 20 mM Hepes-K, 0.1% Tween-20, 8 mM DTT, 50% glycerol; pH 7.2) to a concentration of 1 mg/mL, aliquoted and stored at temperatures between −25 and −18 °C until use.

It should be noted that the Rnh1 preparation used in all comparative experiments was derived exclusively from proteolytic cleavage of the same SUMO-RI batch, because direct independent expression of soluble Rnh1 was not feasible under our conditions. Consequently, the Rnh1 comparator is not an independently produced biological replicate but a derivative of the SUMO-RI construct.

### 2.7. RNA Substrates

Synthetic RNA, serving as an internal control in the SARS-CoV-2/SARS-CoV detection kit (cat. No. R3-P436-S3/9, DNA-Technology LLC, Moscow, Russia), was used as a substrate to evaluate inhibitory activity. In all experiments, aliquots containing a fixed amount of RNA (0.1 µg per reaction) were used.

### 2.8. Inhibitory Activity Studies

The recombinant RIs SUMO-RI and Rnh1, as well as the commercially available Thermo Scientific RiboLock RI with a molecular weight (MW) of 49.6 kDa, and LoRI from GeneQuest LLC (Moscow, Russia) with a MW of 63 kDa, were used for comparative analysis. The stock concentration of all inhibitors was 1 mg/mL, which corresponds to 20.2 µM for Rnh1 and RiboLock as well as 15.9 µM for SUMO-RI and LoRI used for this assay.

The inhibitors are compared at equal amount (e.g., 0.1 µg), despite different MWs without normalization by molar concentration. Indeed, differences in MWs between different inhibitors exist, but they do not exceed 30%. Furthermore, pairwise comparisons of inhibitors with identical MW can be performed. For example, the activity of commercial preparations RiboLock and LoRI can be compared with that of Rnh1 and SUMO-RI, respectively, using the weight values of the quantities used, since RiboLock and Rnh1 have identical MWs of 49.6 kDa, as do LoRI and SUMO-RI, at 63 kDa. Despite these differences in MWs, the experimental design used allowed us to compare the inhibitory activity under equal mass conditions.

The functional activity of the inhibitors was assessed using the real-time PCR (RT-qPCR) protocol of the “SARS-CoV-2/SARS-CoV” kit (DNA-Technology LLC). Reaction mixtures were prepared as follows: 0.5 µL of the enzyme mixture (Taq/RT and 0.1 mg/mL RNase inhibitors) and 10 µL (0.1 µg) of RNA solution. Subsequently, RNase A was introduced at specified amounts (0–20 ng). A total of 15 µL of RT-PCR buffer containing dNTPs and primers (according to the kit’s instructions) was added. Total volume was 26,5 µL. The obtained samples were incubated at 37 °C for 30 min. Molar concentration of inhibitors in the final reaction volume: SUMO-RI, 0.030 µM; Rnh1, 0.038 µM; RiboLock, 0.038 µM; and LoRI, 0.030 µM. After incubation, aliquots were transferred to the final reaction mixture of the diagnostic kit according to the manufacturer’s instructions, and one-step RT-qPCR was performed on a DT-Prime instrument (DNA-Technology LLC, Moscow, Russia). Amplification Program included an initial step at 35 °C for 20 min, followed by initial denaturation at 95 °C for 5 min, and then 50 cycles of denaturation at 94 °C for 15 s and extension at 64 °C for 20 s. Two groups of samples were used as controls: one without the addition of inhibitor but with RNase A (degradation control) and another one without both inhibitor and RNase A (control for RNA integrity and absence of exogenous RNase contamination).

The degree of RNA preservation was assessed by the threshold cycle (Ct) value of the target amplicon detected in the HEX channel. The average Ct values calculated from three technical replicates were used for the analysis. The change in Ct values relative to the control samples served as a measure of the functional protective capacity of the inhibitors. The protective efficacy of RNase inhibitors was assessed by measuring the tCt values of a target RNA amplicon using one-step RT qPCR. The Ct value is the cycle number at which the fluorescence signal from the amplified product exceeds a defined background threshold; it is inversely correlated with the initial amount of intact RNA template in the reaction [22,23,24]. When RNA is subjected to RNase A degradation, the number of amplifiable molecules decreases, leading to a proportional increase in Ct.

### 2.9. Determination of Inhibitor Storage Stability

To assess stability, the recombinant inhibitors LoRI, SUMO-RI, and Rnh1 were stored in the same buffer (50 mM KCl, 20 mM Hepes-K, 0.1% Tween-20, 8 mM DTT, 50% glycerol, pH 7.2). The commercial inhibitor RiboLock was stored in its original storage buffer supplied by the manufacturer. The inhibitors were incubated at various temperatures: +20 °C (room temperature), +2… +8 °C, and −20 °C for 10 days. For analysis, aliquots of the inhibitor stored at different temperatures were taken at different days (days 0, 3, 6, and 10) and 0.5 µL of each aliquot were added to 0.5 µL of an enzyme mixture (Taq/RT), followed by 10 µL (0.1 µg) of an RNA solution. Subsequently, RNase A (10 ng) was introduced into the mixture. The resulting samples were incubated at 37 °C for 30 min. A total of 15 µL of RT-PCR buffer containing dNTPs and primers (according to the kit’s instructions) was added. Total volume was 27 µL. Molar concentration of inhibitors in the final reaction volume: SUMO-RI—0.294 µM, Rnh1—0.373 µM, RiboLock—0.373 µM, and LoRI—0.294 µM. Amplification Program: initial step at 35 °C for 20 min, followed by initial denaturation at 95 °C for 5 min, and then 50 cycles of denaturation at 94 °C for 15 s and extension at 64 °C for 20 s. Residual activity was determined by changes in Ct values.

### 2.10. Determination of the Effective Temperature Range of the Inhibitors

The thermostability of the inhibitors was studied over a temperature range of between 37 and 64 °C. Then a mixture of 5 µg (5 µL of a 1 mg/mL preparation) of the tested inhibitor and 5 ng (0.5 µL of a 10 ng/µL preparation) of RNase A was added to reaction tubes from a “SARS-CoV-2/SARS-CoV” (DNA-Technology LLC) kit containing RNA 0.1 µg per reaction dissolved in the reaction buffer with Taq/RT and RT-PCR analysis was performed. The criterion of inhibitory ability was the absence of amplification or a sharp increase in Ct values, comparable to the control without the inhibitor. Total volume is 31 µL. Molar concentration of inhibitors in the final reaction volume: SUMO-RI—2.56 µM, Rnh1—3.25 µM, RiboLock—3.25 µM, and LoRI—2.56 µM. Amplification Program: initial step at 35 °C for 20 min, followed by initial denaturation at 95 °C for 5 min, and then 50 cycles of denaturation at 94 °C for 15 s and extension at 64 °C for 20 s.

### 2.11. Comparative Kinetic Study

To determine the kinetic parameters and the mode of inhibition, the initial rates of RNA degradation were analyzed at varying substrate concentrations in the absence or presence of different concentrations of each inhibitor. All reactions were carried out in 10× buffer (300 mM Tris-HCl, 50 mM MgCl_2_, 500 mM KCl, pH 7.9–8.0 at 25 °C) using total RNA isolated from a cell line as the substrate. The fluorescent dye Lumiprobe QuDye ssDNA Reagent (Lumiprobe, Hannover, Germany) was used to monitor RNA degradation, since the manufacturer’s website indicates its possible use for RNA detection. Before kinetic experiments, the applicability of Lumiprobe QuDye ssDNA Reagent for detecting RNA under the assay conditions was verified. For this purpose, fluorescence was measured in mixtures containing 4 µL of 10× buffer, 8 µL of Lumiprobe QuDye ssDNA Reagent (0.5% aqueous solution), and RNA at concentrations of 12.5, 50, 100, 200, 400, and 600 pM; nuclease-free water was added to a final volume of 40 µL. The fluorescence signal increased in an RNA amount-dependent manner over the tested range, confirming that the dye provided a reproducible concentration-dependent signal suitable for comparative monitoring of RNA degradation under these experimental conditions. The validation curve is shown in Figure S1. Measurements were performed using a CLARIOstar^®^ Plus microplate reader (BMG LABTECH, Ortenberg, Germany). For each inhibitor (SUMO-RI, LoRI, Rnh1, and RiboLock), the reaction mixture (80 μL final volume) contained a fixed amount of RNase A (5 ng) and increasing concentrations of the inhibitor. The final inhibitor concentrations tested were 0, 200, 225 and 250 pM. Substrate (total RNA) was added at six different concentrations: 12.8, 28.3, 49.7, 98.5, 205.6 and 498.3 pM. The initial velocity (v) was recorded as the change in fluorescence per minute. For the uninhibited control reaction, the initial velocity data were fitted to the Michaelis–Menten equation:

$$v=\frac{V_{max}\cdot [S]}{\left[S\right]+K_{M}}$$

where v is the initial velocity, V_max_ is the maximum reaction velocity, K_M_ is the Michaelis constant, and [S] is the substrate concentration.

In our analysis, we used the standard equation for mixed inhibition:

$$V=\frac{V_{max}\cdot [S]}{[S](1+\frac{\left[I\right]}{K_{iu}})+(1+\frac{\left[I\right]}{K_{ic}})\cdot K_{M}}$$

where V is the rate of the enzymatic reaction, V_max_ is the maximum reaction rate, [S] is the substrate concentration, K_M_ is the Michaelis constant, and [I] is the inhibitor concentration. K_i_, denoted here as K_ic_, represents the inhibition constant for inhibitor binding to the free enzyme, while αK_i_, denoted as K_iu_, represents the inhibition constant for inhibitor binding to the enzyme–substrate complex.

The two inhibition constants were related by:

$$K_{iu}=\alpha \cdot K_{ic}$$

For visualization of inhibitor-containing curves at a fixed inhibitor concentration of 200 pM, apparent kinetic parameters were calculated as:

$$V_{max,app}=\frac{V_{max}}{1+\frac{\left[I\right]}{K_{iu}}}$$

$$K_{M,app}=K_{M}\frac{1+\frac{\left[I\right]}{K_{ic}}}{1+\frac{\left[I\right]}{K_{iu}}}$$

Nonlinear regression analysis was performed using GraphPad Prism, version 10.4.0. Data were fitted to the mixed inhibition model. The quality of the fit was assessed by the coefficient of determination (R^2^) and by examining the residuals. The 95% confidence intervals (CI) for K_i_ were calculated using the profile likelihood method implemented in Prism. To visually compare the relative potency of the four inhibitors under identical conditions, the initial velocities at a fixed inhibitor concentration of 200 pM were plotted as a function of substrate concentration.

### 2.12. Statistical Analysis

All experiments were performed in three technical replicates as independent measurements from the same batch of cells and protein preparations. Data are presented as mean ± SD. Two-way analysis of variance (ANOVA) was used, followed by Tukey’s multiple comparisons test for pairwise comparisons. The family-wise error rate was set at α = 0.05. Adjusted *p*-values are reported. Statistical analyses were performed using GraphPad Prism, 10.4.0.

## 3. Results

Although several types of RNases are involved in RNA processing, the functional activity of the inhibitors developed in this study was evaluated against RNase A, which is widely used as a standard target [1,11].

### 3.1. Structural Modeling of the Complex of SUMO-RI with RNase A Using Boltz-2

**Figure 1:**
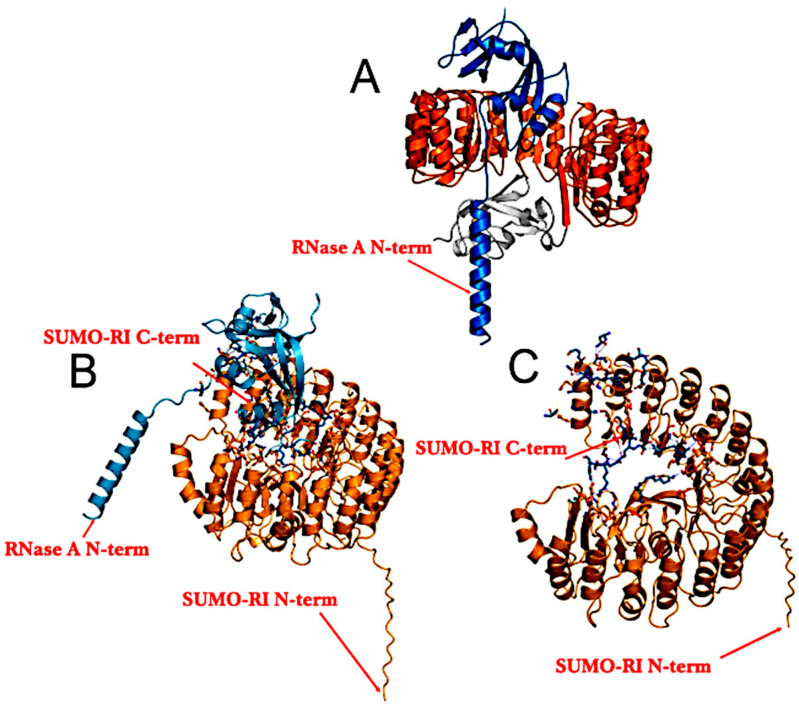
Structural model of the complex of the chimeric SUMO-RI with RNase A, as predicted by Boltz-2. (**A**). Overall view of the complex. RNase A is shown in blue, the SUMO domain in gray, and the RI in orange. (**B**). General view of the complex, demonstrating the predicted binding of RNase A to the C-terminal region of the inhibitor. (**C**). Details of interacting amino acid residues (blue and orange) forming a predicted network of hydrogen bonds, hydrophobic contacts and salt bridges at the inhibitor–RNase interface.

To build the model, chimeric protein SUMO-RI, which included the SUMO domain connected to the N-terminus of the core RNase inhibitor Rnh1, was used. Modeling the complex of SUMO-RI with RNase A using Boltz-2 software demonstrated high prediction reliability: the confidence score was 0.87, with pTM of 0.83 and ipTM of 0.82. In the obtained model (Figure 1), RNase A and both functional domains of SUMO-RI appear to adopt compact stacking with high pLDDT values, while the linker region is characterized by some flexibility.

Analysis of the non-covalent interactions between SUMO-RI and RNase A predicted by PLIP revealed that binding is likely to occur mainly through the C-terminal region of the Rnh1, forming an extensive network of intermolecular contacts (Figure 1). Using the PLIP instrument [19], twenty hydrogen bonds, ten salt bridges, nine hydrophobic contacts and one cation-π interaction were identified.

These data support the feasibility of placing the SUMO domain at the N-terminus, since it does not compete for the RNase A-binding site and, thus, is not predicted to interfere with inhibitory activity. No significant intermolecular contacts were predicted between the SUMO domain and RNase A (only minor hydrogen bond Asp34 SUMO-RI—Asn122 RNase A is observed, with no hydrophobic, π-cation or salt bridge interactions). This result indicates that the SUMO domain can be positioned at the protein’s N-terminus; as it does not compete with the RNase for the binding site, the inhibitory activity is predicted to be not compromised. Preliminary modeling suggests that binding the SUMO domain at the inhibitor’s C-terminus would disrupt the critical contacts between residues and the active center of RNase A [19]. Special attention should be paid to the Tyr537 and Tyr540 residues of the inhibitor, which are predicted to form multiple intermolecular interactions and act as key anchor points of the complex. The strongest hydrogen bond (1.56 Å) is predicted between Asn100 of the RNase and Tyr540 of the inhibitor.

These results suggest that the chimeric architecture of SUMO-RI is likely to retain the inhibitory function of the RI. Key intermolecular contacts with RNase A are predicted to be formed mainly by the RI domain. While the N-terminal SUMO domain remains spatially exposed, it is predicted to be out of the binding region and without creating steric hindrance to the formation of the RI-RNase A complex. These results are consistent with the structural compatibility of the chosen chimeric design with the underlying mechanism of RNase A inhibition.

The overall binding mode predicted by Boltz-2 is broadly consistent with the experimentally determined structures of related RI–RNase complexes. In the crystal structure of the porcine RI–RNase A complex [1], RNase A binds to the C-terminal concave region of the horseshoe-shaped inhibitor, with over 80% of contacting residues contributed by the C-terminal half of RI—a pattern also observed in our computational model. In that experimentally validated structure, the interface buries approximately 2550 Å^2^ of accessible surface area and involves 18 hydrogen bonds and salt links, with Tyr434 of porcine RI serving as a key anchor residue. The analogous residues in our model, Tyr537 and Tyr540 of murine Rnh1, appear to occupy a structurally equivalent position. Notably, the mouse RI–RNase 1 complex (PDB: 3tsr)—the closest structural homologue to the system studied here—buries a comparable interface area of ~2650 Å^2^ and involves 13 hydrogen bonds [2], consistent with the scale of interactions predicted in our SUMO-RI model. Across known RI–RNase complexes, the buried interface area ranges from approximately 2550 Å^2^ in the porcine RI–RNase A complex [1] to ~2908 Å^2^ in the human RI–angiogenin complex [25], with the human RI–RNase 1 complex occupying an intermediate value of ~2800 Å^2^ and forming 19 intermolecular hydrogen bonds [10]—reflecting the generally larger and more electrostatically driven interfaces seen in intraspecies complexes. The number of intermolecular contacts predicted in the SUMO-RI–RNase A model (20 hydrogen bonds, 10 salt bridges, 9 hydrophobic contacts) is broadly consistent in scale with these experimental benchmarks, though a direct quantitative comparison of interface geometry and binding strength requires experimental validation.

### 3.2. Preparation of the Recombinant Inhibitors

**Figure 2:**
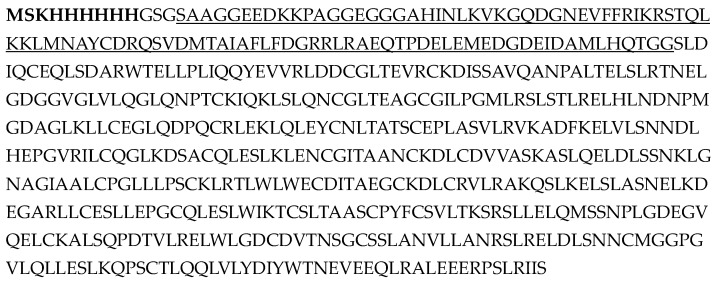
The amino acid sequence of the chimeric protein SUMO-RI. The His-tag is shown in bold; the sequence of the SUMO domain (NCBI Reference Sequence XP_052162678.1) is underlined. RI is mouse Rnh1 (Uniprot accession number Q91VI7).

Based on the results of molecular modeling described above, the chimeric protein with the SUMO domain fused to the N-terminus of RI was prepared by heterologous expression in *E. coli*. To make purification of the target protein easier, a His-tag was added to the N-terminus of the SUMO domain (Figure 2).

**Figure 3:**
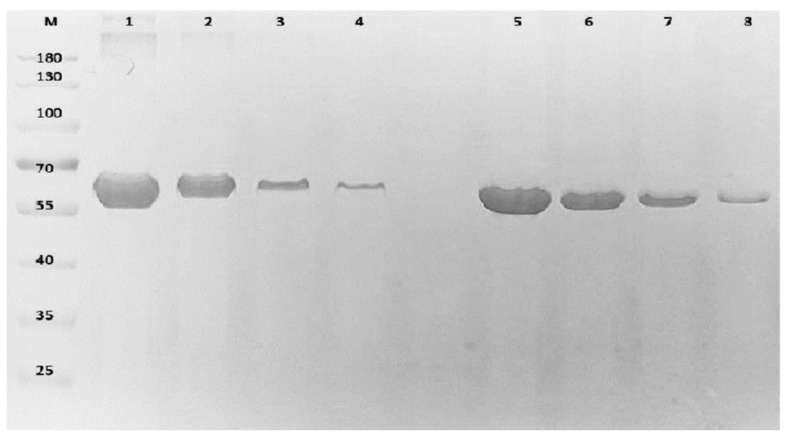
Analysis of purified SUMO-RI with 10% SDS-PAGE. Lane M: MW markers. Lanes 1–4: BSA standard amounts of 5, 2.5, 1.25, and 0.625 μg. Lanes 5–8: final SUMO-RI preparation diluted 4-, 8-, 16-, and 32-fold. SUMO-RI concentration was estimated using the BSA standard curve.

The soluble SUMO-RI chimera was obtained through the expression of the corresponding synthetic gene in *E. coli*. The expression *E. coli* strain was first transformed with the chaperone plasmid pGro7 to enhance the yield of soluble active protein. The produced SUMO-RI was isolated by ammonium sulfate precipitation, metal-affinity and anion-exchange chromatography similarly to the procedure described in [21]. According to gel-electrophoresis, the obtained protein was virtually free from contaminants (Figure 3). The mass of about 60 kDa determined by electrophoresis is close to a theoretical mass of 61.5 kDa for SUMO-RI.

**Figure 4:**
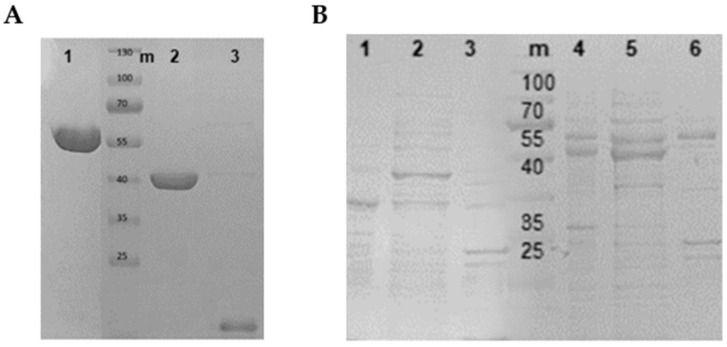
SDS-PAGE (10%) analysis of proteolytic SUMO-RI cleavage products. (**A**) Lane m: MW markers. Lane 1: intact SUMO-RI; lane 2: unbound protein (RI); lane 3: bound protein (SUMO with His-tag). (**B**) Comparison of Rnh1 and SUMO-RI purified by metal-affinity chromatography. Lanes: 1—Rnh1 lysate; 2—Rnh1 debris; 3—Rnh1 eluate; 4—SUMO-RI lysate; 5—SUMO-RI debris; 6—SUMO-RI eluate; m—markers. Both constructs expressed in BL21(DE3)/pGro7 under the same conditions.

To obtain the Rnh1 inhibitor devoid of additional domains, the precise proteolytic cleavage of the SUMO domain was performed using the highly specific peptidase bdSENP1. To separate the cleaved SUMO domain containing the His-tag from the inhibitor, metal-affinity chromatography was used. The inhibitor was not retained on the sorbent and was collected, while the SUMO domain was adsorbed to the column resin. Electrophoretic analysis showed the absence of impurities in the inhibitor obtained (Figure 4A).

The purification provided around 15 mg of chimeric SUMO-RI per liter of bacterial culture. Losses at the stage of proteolytic cleavage of the SUMO domain were equal to around 15% of the initial amount of protein.

Figure 4B shows a side-by-side comparison of the biosynthesis products of the recombinant RNase inhibitors Rnh1 and SUMO-RI obtained by metal-affinity chromatography. The eluate from the Rnh1 expression (lane 3) contained negligible amounts of the target protein, whereas SUMO-RI was readily purified (lane 6), confirming the advantage of the SUMO fusion for soluble production.

### 3.3. Comparative Analysis of the Activity of Different RNase A Inhibitors

**Figure 5:**
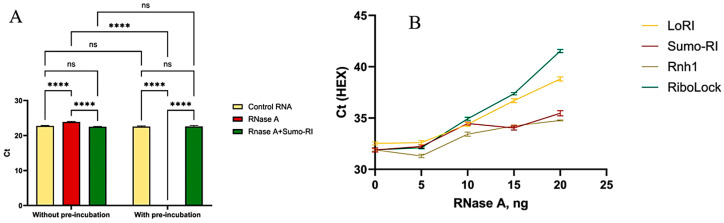
(**A**) Apparent Ct values of varying amounts of RNase A when assayed with 0.1 μg of each inhibitor. Data are presented as mean ± SD, n = 3 technical replicates. Two-way ANOVA; Tukey’s multiple comparisons test (α = 0.05). Without pre-incubation adjusted *p*-values: RNase A vs. control RNA, *p* < 0.0001; RNase A+Sumo-RI vs. control RNA, *p* = 0.2751 (ns); RNase A+Sumo-RI vs. RNase A, *p* < 0.0001. With pre-incubation: RNase A vs. control RNA, *p* < 0.0001; RNase A+Sumo-RI vs. control RNA, *p* = 0.8896 (ns); RNase A+Sumo-RI vs. RNase A, *p* < 0.0001. Control RNA: without vs. with pre-incubation, *p* = 0.1902 (ns). RNase A: without vs. with pre-incubation, *p* < 0.0001. RNase A+Sumo-RI: without vs. with pre-incubation, *p* = 0.5007 (ns). **** *p* < 0.0001; ns—not significant. (**B**) Effect of pre-incubation on the apparent protective activity of RNase inhibitors. Data are presented as mean ± SD, n = 3 technical replicates. Two-way ANOVA followed by Tukey’s multiple comparisons test (α = 0.05) was used to compare all four inhibitors. All pairwise comparisons were statistically significant (adjusted *p* < 0.0001). SUMO-RI and Rnh1 showed lower Ct values under the tested conditions and also differed significantly from each other (*p* < 0.0001). LoRI and RiboLock also differed significantly (*p* < 0.0001). However, because the analysis was based on technical replicates from a single production batch, these differences should be interpreted as assay-level screening observations.

The standard one-step format can yield false-positive results (apparent protection) when inhibitor activity or efficiency are insufficient. In contrast, our pre-incubation approach allows discrimination between inhibitors under conditions where RNA degradation is not counteracted by rapid reverse transcription. To support this rationale, we performed control experiments in which inhibitors were added directly to the one-step RT-PCR reaction mixture without pre-incubation. Under these conditions, differences between inhibitors were abrogated, indicating that the one-step format without pre-incubation does not allow accurate assessment of protective properties (Figure 5A).

Ct values were analyzed under the indicated conditions. Without pre-incubation (standard one-step RT-PCR setup), no difference was observed between samples containing RNase A alone and those supplemented with SUMO-RI. In contrast, when RNA was pre-incubated with RNase A and the inhibitor prior to reverse transcription, a clear reduction in RNase-induced degradation was evident with SUMO-RI. These results suggest that the one-step format without pre-incubation may not reveal differences under these conditions, whereas the pre-incubation approach allows detection of protective activity—though it could also exaggerate differences due to nonstandard conditions.

Two types of controls were used: reactions with no RI (i) and with no inhibitor and no RNase A (ii). In control reactions without an inhibitor, the addition of even trace amounts of RNase A led to RNA degradation and to an increase in Ct values. In the reaction with neither an inhibitor nor RNase A, the presence of endogenous RNases, which could have been in the RNA preparation or introduced during the experiment, was controlled to allow the RNA to remain intact. The combined addition of RNase A and an inhibitor made it possible to quantify the protective properties of each inhibitor via Ct changes relative to the control values.

It should be noted that Ct values obtained via RT-qPCR are a functional indicator of the degree of RNA preservation, rather than a direct biochemical measure of the utility of the interaction of the inhibitor with RNase A or the kinetics of inhibition [24]. The results have therefore been interpreted in terms of the functional protection of RNA in a diagnostic test.

To evaluate the level of RNase A inhibition, various amounts of the enzyme (0, 5, 10, and 20 ng per reaction) were added to the reaction mixture. The functional activity of the inhibitors was assessed using the change in Ct values relative to the control reaction (RNA without the addition of an inhibitor or RNase A).

When added in the amount of 0.1 µg, all the inhibitors protected RNA from degradation by up to 20 ng of RNase A. However, the degree of functional protection differed between inhibitors (Figure 5B). Negative controls where RNase A was added without an inhibitor confirmed the high sensitivity of the assay: the target amplicon was not detected after the addition of only 5 ng of the enzyme.

**Figure 6:**
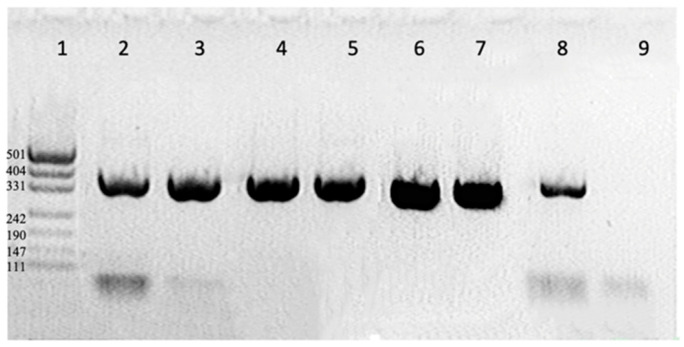
Analysis of RT-qPCR products by electrophoresis in 1.8% agarose gel. For the functional protection of RNA, 0.1 µg of each inhibitor was used during incubation with 20 ng of RNase A, followed by RT-PCR. 1—pUC19/MspI DNA marker; 2 and 3—LoRI; 4 and 5—SUMO-RI; 6 and 7—Rnh1; 8 and 9—RiboLock.

In this assay, SUMO-RI and Rnh1 showed lower Ct values under the tested conditions (Figure 6). However, because the comparisons were based on technical replicates from a single production batch, these differences should be interpreted as assay-level observations rather than as definitive evidence of superior performance. Notably, the inhibitors that were added at lower molar concentrations showed a stronger RNA protection tendency than RiboLock. Therefore, the difference in molarity, while not very large, does not compromise the validity of this qualitative conclusion.

### 3.4. Evaluation of the Inhibitors’ Stability

To assess the stability of the inhibitors, inhibitor samples were incubated at various temperatures: +20 °C, +4 °C, and −20 °C. The preparations were stored for ten days with activity control measurements on days three, six, and ten. Before the analysis, each inhibitor was mixed with 10 ng of RNase A, after which each mixture was added to a ready-made reaction mixture for one-step RT-PCR containing primers, reverse transcriptase and Taq-polymerase. These results were compared with measurements performed immediately after sample preparation (day zero).

**Figure 7:**
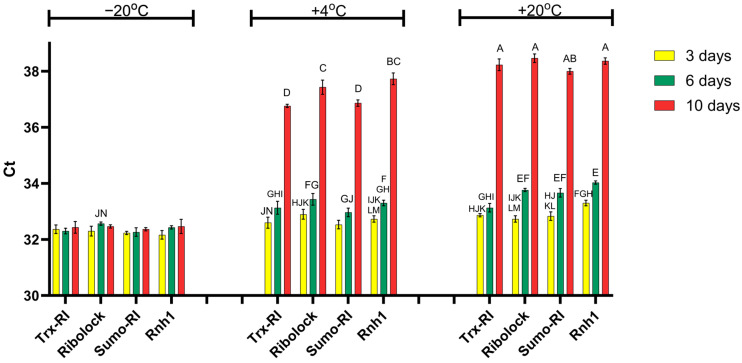
Apparent Ct values obtained using 10 ng of RNase A after three, six, and ten days of incubation with the inhibitors at −20 °C (**left**), +4 °C (**center**), and +20 °C (**right**). Data are presented as mean ± SD, n = 3 technical replicates. Two-way ANOVA followed by Tukey’s multiple comparisons test were performed separately for each temperature. Groups sharing the same letter are not significantly different; groups with different letters differ significantly. Full pairwise adjusted *p*-values are provided in the source data (Table S1).

Analysis of the RT-PCR data showed that storage temperature affected the residual protective activity of the inhibitors. At −20 °C, the inhibitors retained their activity over the 10-day observation period. At +4 °C, the preparations remained largely active, but after 10 days a small Ct shift was observed, indicating a minor decrease in functional activity. This effect was less pronounced than at +20 °C, where all inhibitors showed a clear reduction in protective activity after 10 days. Therefore, Figure 7 indicates that activity loss is not restricted to room temperature storage, although it is substantially stronger at +20 °C than at +4 °C.

### 3.5. Determination of the Effective Temperature Range

To determine the effective temperature range of RNase A inhibition, the effects of each inhibitor were studied at temperatures from 37 to 64 °C. Each inhibitor (5 µg) was assayed with RNase A (5 ng) and these mixtures were added to RNA dissolved with Taq/RT in the reaction buffer. The samples were incubated at various temperatures for 30 min, after which RT-PCR analysis was performed.

**Figure 8:**
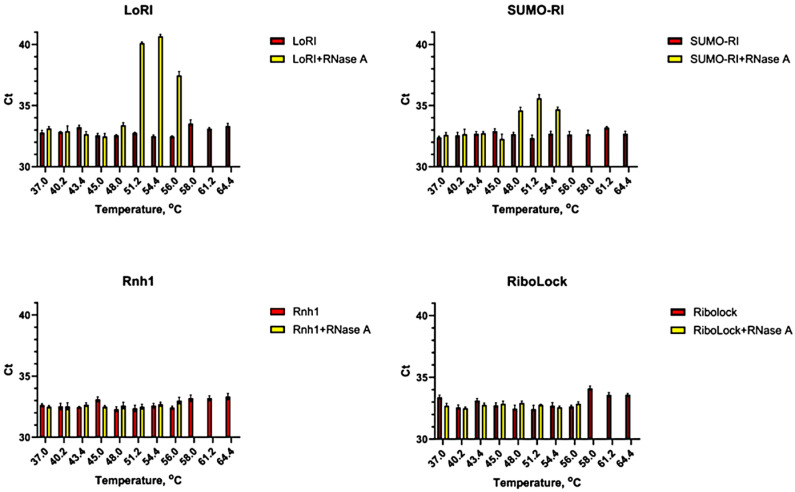
Ct values obtained by RT-PCR after incubation of samples containing 5 ng RNase A and 5 µg of inhibitor at different temperatures. Data are presented as mean ± SD, n = 3 technical replicates.

Statistical analysis confirmed that, within the temperature range where inhibitory activity was still detectable, the inhibitors differed in their protective capacity (Figure 8). At 37–56 °C, LoRI showed significantly weaker protection than Rnh1, SUMO-RI, and RiboLock, whereas no significant differences were observed among Rnh1, SUMO-RI, and RiboLock. At t ≥ 58 °C and above, no inhibitor retained detectable protective activity, and pairwise comparisons were no longer significant.

The effective temperature range analysis showed that SUMO-RI retained activity under standard assay conditions but began to lose activity above approximately 47 °C. SUMO-RI and LoRI lost inhibitory capacity earlier than Rnh1 and RiboLock, whereas Rnh1 and RiboLock retained pronounced protective properties up to approximately 56 °C. This indicates that SUMO fusion improves the technological profile of the inhibitor but is associated with a moderate decrease in thermostability.

### 3.6. Comparative Kinetic Study

**Figure 9:**
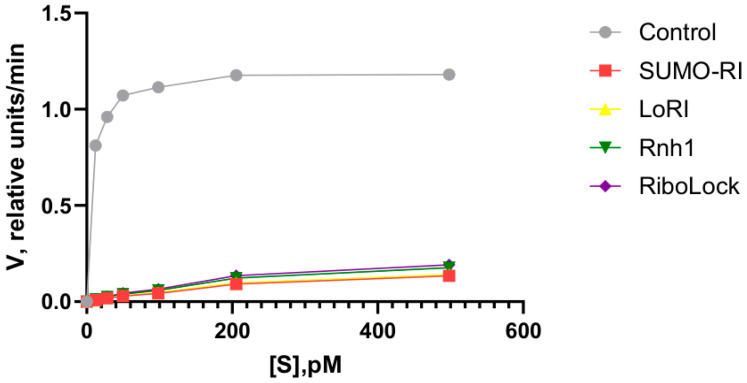
Kinetic analysis of RNase A inhibition by inhibitors using nonlinear regression (mixed inhibition model). The control curve corresponds to the reaction without inhibitor. The inhibitor-containing curves were obtained at a fixed concentration of 200 pM for each RNase inhibitor.

To determine the type of inhibition and to calculate the inhibition constants, we analyzed the initial velocity of RNA degradation as a function of substrate concentration [S] in the presence of different concentrations of each inhibitor. Prior to kinetic analysis, the fluorescence response of Lumiprobe QuDye ssDNA Reagent to RNA was verified under the assay conditions. The fluorescence signal increased with increasing RNA amount in the range of 12.5–600 pg, supporting the use of this readout for comparative monitoring of RNA degradation. Nonlinear regression was performed using the mixed inhibition model in GraphPad Prism 10 (Figure 9). This model assumes that the inhibitor can bind to both the free enzyme and the enzyme–substrate complex, with a factor α modifying the affinity of the substrate when the inhibitor is bound [26]. The global fit of the data for each inhibitor yielded the following inhibition constants (K_ic_): SUMO-RI—0.765 pM (95% CI: 0.71–0.82), LoRI—0.825 pM (0.78–0.88), Rnh1—1.059 pM (0.98–1.14), and RiboLock—1.199 pM (1.11–1.29). In the control sample without inhibitor, Ki, K_ic_, K_iu_, and α were not determined, since no inhibitor was present. The maximum reaction velocity was 1.20 relative units/min, and the Michaelis constant was 6.3 pM and characterized by R^2^ = 0.994. For SUMO-RI, the coefficient α was 51 and the calculated inhibition constant for inhibitor binding to the enzyme–substrate complex was K_iu_ = αK_ic_ = 39.02 pM. At an inhibitor concentration of 200 pM, the apparent maximum velocity was 0.232 relative units/min, the apparent Michaelis constant was approximately 352 pM and the goodness of fit was R^2^ = 0.989. For LoRI the coefficient α was 49 and the calculated K_iu_ = αK_ic_ value was 40.43 pM. The apparent maximum velocity was 0.239 relative units/min, the apparent Michaelis constant was approximately 337 pM, and R^2^ = 0.987. For Rnh1 the coefficient α was 50, and the calculated K_iu_ value was 52.95 pM. The apparent maximum velocity was 0.297 relative units/min, the apparent Michaelis constant was approximately 328 pM, and R^2^ = 0.991. For RiboLock the coefficient α was 48 and the calculated K_iu_ = αK_ic_ value was 57.55 pM. The apparent maximum velocity was 0.316 relative units/min, the apparent Michaelis constant was approximately 310 pM, and R^2^ = 0.985.

Among the tested inhibitors, SUMO-RI showed the lowest fitted K_ic_ value and the lowest calculated K_iu_ value. This indicates the strongest apparent interaction with free RNase A and the lowest residual velocity under the tested assay conditions. However, because K_iu_ remained substantially higher than K_ic_, this result should not be interpreted as preferential binding of SUMO-RI to the enzyme–substrate complex. K_iu_ values were substantially higher than the corresponding K_ic_ values, and the inhibitors bound more strongly to free RNase A than to the enzyme–substrate complex. Therefore, the kinetic behavior is best described as apparent mixed inhibition with a dominant competitive component, rather than preferential binding to the enzyme–substrate complex. To compare the relative potency of the four inhibitors under identical conditions, we plotted the reaction velocity versus substrate concentration at a fixed inhibitor concentration of 200 pM (Figure 9).

Although the kinetic parameters were derived from globally fitted models, direct comparison between SUMO-RI and Rnh1 assumes that the cleavage process does not alter the intrinsic properties of the Rnh1 domain.

The velocity traces were consistently higher for inhibitors with higher K_i_ values, confirming the order of inhibitory efficacy: SUMO-RI (lowest velocity) > LoRI > Rnh1 > RiboLock (highest velocity). These data suggest that, within this single-batch screening assay, SUMO-RI showed the lowest fitted K_ic_ value and the lowest initial velocities at 200 pM inhibitor concentration. This observation supports preserved inhibitory activity of the SUMO-RI construct under the tested conditions, but should not be interpreted as a definitive ranking of inhibitor performance.

These fitted parameters should not be overinterpreted as absolute ranking; rather, they support the conclusion that SUMO-RI retains inhibitory activity comparable to some commercial preparations under the conditions tested. Hence, independent replication is needed to assess generalizability.

## 4. Discussion

Our structural modeling data obtained using the advanced Boltz-2 algorithm confirm a critical design premise: the spatial placement of the SUMO module does not occlude the interface where the Rnh1 domain binds to RNase A. The algorithm predicted a high-confidence complex where key intermolecular interactions are maintained exclusively by the inhibitor, validating the structural neutrality of N-terminus fusion. This finding is significant as it suggests that the SUMO domain functions primarily as an autonomous folding and solubility enhancer without disturbing the intricate, cysteine-rich architecture essential for RNase inhibitors. Notably, this intricate cysteine network has been shown to contribute to intracellular redox homeostasis [4], underscoring its functional importance beyond mere structural integrity. The minimal involvement of the SUMO domain in the binding interface, limited to two peripheral contacts, underscores the success of this chimeric design in avoiding steric interference—a common pitfall in fusion protein engineering.

The protective efficacy of RNase inhibitors was assessed by measuring the Ct values of a target RNA amplicon using one-step RT-qPCR. This method has been employed in several previous studies to compare the protective capacity of recombinant RNase inhibitors, demonstrating its utility as a reliable, application-oriented endpoint [12,21]. This relationship has been extensively validated in diagnostic and research settings, where Ct shifts are routinely used to quantify RNA integrity and to evaluate the performance of RNase inhibitors under conditions that mimic real world sample handling [12,24]. Accordingly, the Ct values obtained herein serve as a quantitative measure of functional RNA protection, rather than as a direct surrogate for inhibitor–enzyme binding kinetics. Inhibitor activity was assessed under conditions under which RNA was pre-incubated with RNase A and with the tested inhibitors prior to RT-PCR. This approach was deliberately adopted because in routine applications (e.g., RNA isolation, storage, freeze–thaw cycles), RNA is often exposed to RNase contamination before reverse transcription or RT-PCR setup. Consequently, the most vulnerable stage is the period prior to reverse transcription. We designed conditions that allow evaluation of how effectively an inhibitor protects the template during this critical window, while also challenging the system by combining RNase activity with thermal stress. Under the pre-incubation scheme, RNA degradation can occur before amplification begins, thereby creating more stringent conditions for inhibitor testing. If an inhibitor effectively protects RNA under these conditions, it is highly likely to be effective under standard RT-PCR or reverse transcription setups. In the classic one-step RT PCR format, reverse transcription initiates immediately after mixing the components. In this scenario, cDNA synthesis may outpace RNA degradation, potentially masking incomplete inhibitor efficacy.

Functionally, both the chimeric SUMO-RI and its cleaved counterpart, Rnh1, proved effective in protecting RNA from degradation under RT-qPCR conditions, which is widely used in molecular biology and a cornerstone assay in molecular diagnostics. Within our experimental assays, their performance was comparable with commercially available inhibitors. This indicates that SUMO fusion and the subsequent cleavage process do not introduce deleterious modifications that would compromise the inhibitor‘s ability to recognize and neutralize RNase A. The observed functional parity between SUMO-RI and Rnh1 reinforces the conclusion that the SUMO tag does not present a direct functional advantage nor disadvantage to inhibition per se. However, given that the Rnh1 used here was obtained by cleavage of SUMO-RI, we cannot rule out that the cleavage process itself might subtly affect activity. Therefore, the observed functional parity should be considered supportive but not definitive proof of equivalence.

The stability assessment reveals a nuanced trade-off inherent to the SUMO fusion strategy. SUMO-RI showed a favorable technological profile in this production batch, including improved soluble recovery and storage behavior under the tested conditions. This aligns with the well-documented role of SUMO in promoting the proper folding and solubility of recombinant proteins in *E. coli*. However, this advantage comes with a compromise in thermostability as SUMO-RI became less effective at temperatures approximately above 47 °C, slightly earlier than the Rnh1 and the commercial RiboLock inhibitor. The reduced thermal stability of the chimeric form may be attributed to the added structural domain, which could alter the protein’s global stability or dynamics under thermal stress. Importantly, this temperature threshold partially overlaps with the reverse transcription step in one-step RT-qPCR protocols. While this factor may cause problems for protocols employing higher incubation temperatures, it did not impair functionality at the physiological conditions of 37 °C, used in this and many other studies. Therefore, the trade-off appears acceptable within the context of typical in vitro diagnostic workflows, where storage stability and production yield are often paramount.

A relevant study by Guo et al. [17] showed that murine RI fused to several N-terminal partners, including SUMO, was inferior to MBP in terms of soluble protein yield. However, the novelty of our work is defined by fundamental differences. First, Guo et al. aimed to maximize the yield of native RI after tag removal, whereas we deliberately characterized SUMO-RI as a standalone chimeric inhibitor suitable for use without SUMO domain removal—a different engineering strategy. Second, we provide the first detailed structural modeling (Boltz-2) of the SUMO-RI complex with RNase A, showing that the SUMO domain does not participate in target binding and does not create steric hindrance, thus rationalizing the use of SUMO-RI as a fully functional inhibitor without tag removal. Third, our study includes a comprehensive evaluation of technological properties—storage stability, effective temperature range, and direct comparison with commercial inhibitors under real-world diagnostic workflows—aspects not addressed by Guo et al. [17]. Moreover, our approach leverages modern SUMO-specific proteases (e.g., bdSENP1) and improved purification strategies, providing a more flexible platform: SUMO-RI can be used either in its chimeric form or as a native inhibitor after cleavage. A direct comparison of the yields reachable with the two different approaches is complicated by differences in expression strains, chaperone use, and purification protocols. Indeed, Guo et al. did not employ chaperone co-expression and optimized their process for tag removal, whereas we deliberately retained the SUMO tag. Therefore, the lower soluble yield of SUMO-RI compared to MBP-RI in that study does not contradict our finding that SUMO-RI is a functional and stable chimeric reagent—the two studies pursuing different engineering goals. Therefore, our work presents a new engineering concept in which SUMO fusion serves not only as a solubilizing tag but also as an integral part of the final functional product with a distinct profile of technological properties.

The seminal work by Kobe and Deisenhofer [1] established that the RI blocks the RNase A active site via steric occlusion—a mechanism often described in classical terms as ‘competitive’. In our study, kinetic analysis revealed an apparent mixed inhibition pattern for all tested inhibitors. However, the calculated α values were substantially greater than 1, resulting in K_iu_ values approximately 48–52 times higher than K_ic_. This indicates that the inhibitors bind more strongly to free RNase A than to the enzyme–substrate complex. Consequently, the observed inhibition pattern should be interpreted as mixed inhibition with a dominant competitive component rather than preferential binding to the enzyme–substrate complex. This interpretation is consistent with the crystal structure of the RI–RNase A complex [1]. The finite K_iu_ values (39–58 pM) suggest that even when RNA is bound, the inhibitor retains some affinity for the complex. This may reflect residual conformational flexibility of the RI–RNase interface or non-competitive contributions from peripheral contacts. To our knowledge, such comparative kinetic analysis across multiple RI variants (chimeric, tag-free, and commercial) has not been previously reported.

Taken together, these findings define both the potential and the limitations of the SUMO-RI engineering strategy. The comparison with previous work indicates that SUMO should not be considered only as a removable solubilization tag; in the present construct, it can function as part of the final chimeric inhibitor. At the same time, all functional, stability, and kinetic comparisons were performed using technical replicates from a single production batch. Therefore, the adjusted pairwise *p*-values should be interpreted as reflecting assay-level differences under the tested conditions rather than definitive batch-independent differences between inhibitor preparations. Independent production batches and biological replicates will be required to confirm the generalizability of these findings and to define the activity spectrum against RNases other than RNase A.

## 5. Conclusions

This study demonstrates that N-terminal fusion of SUMO to murine Rnh1 yields a chimeric RNase A inhibitor, SUMO-RI, with preserved structural and functional integrity. Computational modeling indicated that the SUMO domain does not interfere with the Rnh1–RNase A binding interface, and functional assays confirmed that SUMO-RI protects RNA under RT-qPCR conditions. The SUMO fusion improved soluble production and supported favorable storage behavior, but this technological advantage was accompanied by reduced thermostability above approximately 47 °C compared with tag-free Rnh1. Therefore, SUMO-RI should be regarded as a rational engineering solution for applications where soluble recovery, storage stability, and functionality under standard diagnostic conditions are more important than high-temperature robustness.

Overall, the present work provides proof-of-concept evidence that N-terminal SUMO fusion can be used to obtain a soluble and functionally active RNase A inhibitor preparation; however, definitive comparison with commercial inhibitors and batch-independent performance ranking will require independent production batches and biological replication.

## Data Availability

The original contributions presented in this study are included in the article. Further inquiries can be directed to the corresponding author.

## Supplementary Materials

The following supporting information can be downloaded at: https://www.mdpi.com/article/10.3390/cimb48060637/s1.

## Abbreviations

The following abbreviations are used in this manuscript:

RNA	Ribonucleic acid
RNase	Ribonuclease
RT-PCR	Reverse transcription-polymerase chain reaction
RI	Ribonuclease inhibitor
SUMO	Small Ubiquitin-like Modifier
BSA	Bovine albumin concentration
RT-qPCR	Reverse transcription-quantitative polymerase chain reaction
Ct	Threshold cycle
