# Supplementary material for: Development of Chimeric Ribonuclease A Inhibitor for Molecular Biology Applications: SUMO Fusion as an Engineering Strategy
Source: Curr Issues Mol Biol. 2026 Jun 18;48(6):637. doi: 10.3390/cimb48060637 (PMC13298494; doi:10.3390/cimb48060637)
Supplement: Supplementary file 1 [file cimb-48-00637-s001.zip › cimb-4347840-supplementary.pdf]

Supplementary

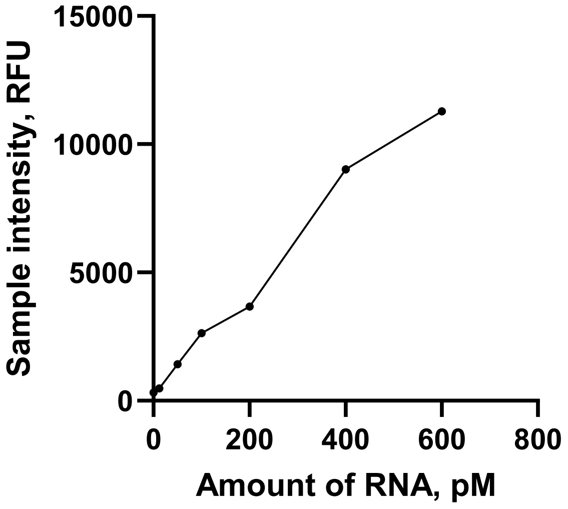

Figure S1. Fluorescence response of Lumiprobe QuDye ssDNA Reagent to increasing RNA amounts under the assay conditions. The fluorescence signal increased with increasing RNA amount, supporting the use of this reagent as an operational comparative readout for RNA degradation under the selected assay conditions. Data are presented as mean ± SD from three technical replicates performed within a single experiment.
